# Challenges in the Borderline Personality Disorder diagnostic in clinical practice in community: results of a pilot study

**DOI:** 10.1192/j.eurpsy.2023.2059

**Published:** 2023-07-19

**Authors:** E. Chumakov, D. Charnaia, N. Petrova

**Affiliations:** 1 Department of Psychiatry and Addiction, Saint-Petersburg State University, Saint-Petersburg, Russian Federation

## Abstract

**Introduction:**

Borderline personality disorder (BPD) has a significant presence in outpatient psychiatric practice worldwide, but data on the clinical features of patients with BPD in Russia are limited. Clinicians experience a number of difficulties in diagnosing BPD, which is also due to the high comorbidity of BPD with other mental disorders (affective, anxiety, other personality and addictive disorders).

**Objectives:**

The aim of this pilot study was to investigate clinical characteristics of mental health care for patients с BPD in real clinical practice in community in Saint-Petersburg, Russia.

**Methods:**

Fifty patients (72% female; n=36; mean age 22.4±4.3) who were treated in an outpatient community care were included in the study. Diagnosis was made according to the ICD-10 criteria (F60.31), as it does in clinical practice in Russia. Research methods included a clinical-catamnestic method.

**Results:**

The age of onset of BPD symptoms was 14.9±2.7. It was found that 50% of patients had previously received inpatient (20%, n=10) and outpatient (30%, n=15) psychiatric care unrelated to the current mental condition, that is, not due to the BPD. 16% of patients (n=8) referred for the first time for psychiatric care in adolescence. The vast majority of patients (86%; n=43) were not diagnosed with BPD when they first consulted a psychiatrist. Prior to the diagnosis of BPD, patients were diagnosed with mental disorders due to organic brain injury (4%), affective disorders (44%), schizophrenia spectrum disorders (12%), anxiety disorders (20%) and other personality disorders (6%). On average, it took 7.5±4.0 years from the date of first psychiatric assessment before the diagnosis of BPD was confirmed. At the time of inclusion in the study, patients were diagnosed with the following comorbid mental health conditions: affective disorders (12%), anxiety disorders (6%), eating disorders (4%), and addictive disorders (2%).

**Conclusions:**

The hypodiagnosis of BPD in the early stages of the disorder has been identified, making it challenging to obtain high quality mental health care in a timely manner. The frequent comorbidity between BPD and other mental disorders has been confirmed. The study demonstrates the relevance of introducing programs (including education) to improve the diagnosis and study of comorbidity of BPD in real clinical practice. The pilot study results provide the basis for a project to investigate approaches to differential diagnosis and evaluation of treatment strategies for patients with BPD.

**Disclosure of Interest:**

None Declared

